# TCM syndrome differentiation and treatment of narcolepsy based on neurobiological mechanism: A review

**DOI:** 10.1097/MD.0000000000032025

**Published:** 2022-12-09

**Authors:** Zhao Liu, Ruiqian Guan, Limin Pan

**Affiliations:** a Heilongjiang University of Traditional Chinese Medicine, Harbin, Heilongjiang Province; b Second Affiliated Hospital of Heilongjiang University of Traditional Chinese Medicine, Harbin, Heilongjiang Province; c The First Affiliated Hospital of Heilongjiang University of Traditional Chinese Medicine, Harbin, Heilongjiang Province.

**Keywords:** acupuncture treatment based on syndrome differentiation, and other treatment methods, DQBI*06:02 T cell TCM treatment based on syndrome differentiation, massage, narcolepsy or exin neuron NT1, NT2

## Abstract

Narcolepsy is a relatively rare brain disorder caused by the selective loss of orexin neurons. Narcolepsy is divided into Narcolepsy Type 1 (NT1) and Narcolepsis Type 2 (NT2). The pathogenesis of NT1 has been well established due to the severe loss of orexin neurons, while NT2 is still poorly understood, and little is known about its underlying neurobiological mechanisms. human leukocyte antigen alleles have been found to strongly influence the development of narcolepsy, with more than 90% of NT1 patients carrying the human leukocyte antigen II allele DQB1*06:02. In addition to the genetic evidence for the DQBI*06:02 allele, some other evidence suggests that a T cell-mediated immune mechanism destroys the orexin neurons of NT1, with CD4 + T cells being key. For this disease, traditional Chinese medicine (TCM) therapy has its own characteristics and advantages, especially the combination of acupuncture and medicine in the treatment of this disease in TCM, which has made considerable and gratifying progress. The purpose of this review is to introduce the frontier progress of neurobiology of narcolepsy, and to explore the syndrome differentiation and treatment of narcolepsy with the combined use of TCM and Western medicine combined with TCM.

## 1. Introduction

Although narcolepsy was described in the literature before the 19th century, it was not until the end of the 19th century that Westphal, Gelinan and Fischer published 3 articles^[1]^ that the 2 main manifestations of narcolepsy were first clearly proposed. One is an irresistible or compulsive excessive daytime sleepiness, and the other is a transient loss of muscle tone or control triggered by emotion and accompanied by a state of consciousness preservation.^[2]^ These observations were confirmed by later researchers.^[1]^ Since then, however, it has been widely accepted that narcolepsy is a rare and nonspecific manifestation of other diseases.^[3]^ By 1960, however, the main features of narcolepsy had been accurately described in a large series of cases, and the specificity of the disease was clearly established.^[3]^

Classic narcolepsy, now called Narcolepsy Type 1 (NT1), is characterized by the presence of convulsive symptoms or a deficiency of orexin.^[4]^ In contrast, narcolepsy without convulsive symptoms or orexin deficiency, now known as Narcolepsy Type 2 (NT2), remains little known. Although the presence of mild and incomplete narcolepsy manifestations (“narcolepsy borderline”) is generally accepted, their inclusion in the diagnostic spectrum remains controversial.^[5]^

Our understanding of the clinical presentation, etiology, pathophysiology, diagnosis, and management of narcolepsy has advanced considerably over the past 20 years.^[3]^ In 1998, 2 groups independently discovered orexin A and orexin B, small neuropeptides produced only by neurons in the lateral hypothalamus. Soon after, researchers found that narcolepsy, caused by severe orexin neuron loss, resulted in low orexin levels in the brain and cerebrospinal fluid (CSF).^[6]^ This discovery led to the recognition of 2 types of narcolepsy: NT1 and NT2. Typical NT1 is characterized by prolonged somnolence plus convulsions, and the CSF of this disorder has very low or barely detectable orexin levels due to severe loss of orexin neurons. The symptoms of NT2 are generally less severe, and 90% of patients have normal orexin levels in the CSF. NT2 affects up to half of narcolepsy patients^[5]^ and may also be caused by partial loss of orexin neurons, but little is known about its underlying neuropathology.^[5]^

The clear association of NT1 with the selective loss of orexin neurons has triggered great progress in our understanding of narcolepsy, but major problems remain. This article aims to describe the latest research progress in the neurobiological mechanism of narcolepsy and explore the possibility of the combined use of traditional Chinese medicine (TCM) and Western medicine in the treatment of narcolepsy combined with TCM.

### 1.1. Cognition of narcolepsy in TCM

The earliest description of narcolepsy in TCM can be found in Miraculous Pivot: “Those who lie down suddenly…pathogenic qi stays in upper jiao pattern…Wei-defensive qi stays in Yin for long time and does not work."^[7]^ Qi is the intangible, high-mobility nutritive substance that maintains vital activities. Pathogenic qi is opposite to healthy qi, it is a collective term for all pathogenic factors. Upper Jiao pattern is characterized by fever, sweating, coughing and panting. Unconsciousness and delirium may also be present. Wei-defensive Q is opposite to the Ying-nutrient qi. It travels outside the vessels and is transformed from the nutrients of water and food. It is worth noting that there is no name of “narcolepsy” in TCM, but modern physicians classify it into traditional pattern syndromes such as “excessive sleep,” “lethargy,” “vertigo,” and “epilepsy.”

Pattern Syndrome is a pathological summarization of the disease location, nature, severity, and prognosis in a certain stage. Five zang organs is a collective term for the 5 internal organs—the heart, liver, spleen/pancreas, lung, and kidney. Treatment based on pattern identification is the whole process of applying theories, principles, prescriptions, and medicines to clinical treatment. Pattern differentiation aims to analyze the signs and symptoms collected by the 4 examination methods according to the fundamental theories of the 8 principles, zang-fu organs, etiology, and pathogenesis.

Treatment refers to specific therapeutic methods directed at the differentiated pattern, and has not yet formed a unified understanding of the etiology and pathogenesis of this disease, but modern physicians have combined the research progress of Western medicine. Professor Wang Xuefeng holds that the basic pathogenesis of this disease is Yang deficiency with yin excess, disharmony between yin and yang, and that the main cause of this disease is dampness blocking Wei-defensive yang pattern.^[7]^ In the view of TCM, Yin-yang is a concept defined as the opposing but complementary qualities of interrelated entities/phenomena in the natural world. Yang deficiency with yin excess is a mutual restraint between yin and Yang maintaining the relative Yin-Yang equilibrium. The failure of yang to restrain yin may cause relative yin excess. Damp phlegm pattern is characterized by cough with easy expectoration of profuse, sticky phlegm, limb heaviness, dizziness, tinnitus, chest stuffiness, and tightness, low food intake, greasy taste and foreign body sensation in the throat. Often results from the internal buildup of phlegm dampness. Dampness blocking Wei-defensive Yang pattern is characterized by fever, aversion to cold, mild sweating, headache, lassitude, chest tightness, poor appetite, and absence of thirst. The tongue coating is thin, white, or greasy. The pulse is delayed. This pattern often occurs when dampness blocks the flow of Wei-defensive qi.

Qiu Changlin believed that the basic pathogenesis of this disease is Spleen qi deficiency pattern. In TCM’s perspective, spleen system is a functional system composed of the spleen, stomach, muscle, lips, mouth, and spleen meridian. Spleen qi means that qi stored in the spleen. It is the driving force of the physiological activity of the spleen. Spleen qi deficiency pattern is characterized by low food intake, abdominal distension that aggravates after eating food, loose stools, and lassitude. The tongue is pale with a white coating. The pulse is slack and weak. The spleen governs transportation and transformation, and ascends the nutrients, and the emotion of the spleen is overthinking. Deficiency of spleen qi leads to weakness in transportation and transformation, and the spleen ascends the spleen ascends the nutrients.

If the spleen ascends the nutrients cannot ascend, the phlegm will stop gathering, which will hinder the middle jiao and brain. Deficiency of spleen qi affects the mind, and excessive thinking affects the mind, resulting in deficiency of the heart and spleen pattern. Deficiency of the heart and spleen pattern leads to lassitude and thinking of lying down, lying down without getting up, sleeping and dreaminess.^[8]^ It should be noted that the middle jiao pattern is characterized by fever, thirst, abdominal fullness, and constipation. Alternatively, subjective feverish sensation without elevated temperature, nausea, vomiting, stomach stuffiness, and loose stools. Often occurs when pathogenic warm heat affects the spleen and stomach and transforms into dryness or dampness. The spleen ascends the nutrients, means that the spleen qi ascends and distributes nutrients to the heart and lungs, and maintains the normal position of the internal organs. Deficiency of the heart and spleen pattern is characterized by palpitations, lassitude, low food intake, abdominal distension, and loose stools. The tongue is pale. The pulse is weak. This pattern often occurs when heart, blood, and spleen (yang) qi become deficient.

In addition, Zheng Zhongqi^[9]^ believed that the pathogenesis of this disease was the disadvantage of Shaoyang Cardinal. Shaoyang pattern is characterized by a bitter mouth, dry throat, dizziness, blurred vision, fever alternating with aversion to cold, fullness in the chest and subcostal region, no desire to eat or drink, restlessness, and vomiting. The pulse is wiry. This pattern often occurs when pathogenic factors affect the qi flow of Shaoyang meridians.

Zhang Hongbin believes that this disease is kidney essence deficiency pattern.^[9]^ But all of which are the various visions of various physicians. Kidney system is a functional system composed of the kidney, urinary bladder, bones, hair, ears, urethra, anus, and kidney meridian. The kidney stores and secures the essence of the human body. Means that all tangible nutrients of the human body. Of essence. It can also specifically refer to the kidney essence. Kidney essence deficiency pattern is characterized by delayed growth in children, decreased reproductive function, premature aging, tinnitus, hair loss, loose teeth, and poor memory. This pattern often results from kidney essence deficiency. Marrow is an extraordinary organ. A collective term for brain marrow, spinal cord, and bone marrow. Marrow is transformed from kidney essence. Kidney marrow deficiency pattern is characterized by delayed growth and development. Delayed healing of bone fractures, low back/knee soreness and weakness, dizziness, tinnitus, poor memory, and dementia. This pattern often results from kidney essence/marrow deficiency.

However, after careful exploration, it can be considered that the deficiency pattern in essence and the excess pattern on the surface. Excess pattern is characterized by high fever, thirst, chest tightness, panting, rapid breathing, phlegm sounds in the throat, restlessness, delirium, abdominal distension/pain/tenderness, and scanty, dark-yellow urine or hesitant, painful urination. The tongue is rough with a dry, yellow coating. The pulse is replete and forceful. Contributing factors may include 6 pathogenic factors, parasites, or dysfunction of the zang–fu organs (subsequent food stagnation, retention of phlegm/fluid/water/dampness, and blood stasis). Deficiency pattern: it is characterized by general weakness, a weak pulse, and abdominal softness. Often results from insufficiency of qi, yin, yang, or blood. Therefore, under the guidance of this principle, this article explores the possibility of treating this disease by the combined use of TCM and Western medicine based on the neurobiological mechanism of Western medicine.

### 1.2. Neurobiological basis

#### 1.2.1. The function of or’exin neurons.

Orexin neurons regulate many functions, chief among them the physiological functions of stabilizing wakefulness and coordinating rapid eye movement (REM) sleep.^[10]^ In rodents, orexin neurons are active during wakefulness, especially during increased exercise or motivated behavior; in contrast, these neurons were relatively inactive during quiet wakefulness, as well as during sleep. According to related studies, intracerebroventricular injection of orexin A or orexin agonists in rodents can promote wakefulness and strongly inhibit REM sleep for several hours.^[11]^ This is most likely done by stimulating neurons in the basal forebrain and monoaminergic nuclei to promote arousal and inhibit REM sleep. In the transition to sleep, GABAergic neurons in the lateral preoptic area and median preoptic area may inhibit orexin neurons and many other wakefulness-promoting neurons, enabling animals to maintain sleep.^[12]^

How Orexin a produces such a long arousal period is unknown, and little is known about orexin neurons in humans, but orexin A may be present in the extracellular space for a long time, while orexin B may have a short half-life.^[13]^ Orexin peptide may automatically activate orexin neurons via OX2R. Therefore, once these cells fire, they may remain active for a long time, contributing to long-term maintenance of wakefulness.^[14]^ However, this view of automatic firing is controversial, as some researchers have reported that orexin neurons do not express the orexin receptor.^[14]^ At present, little is known about the firing pattern of orexin neurons, and more long-term recordings are needed.^[15]^ It should be noted that the conditions triggering orexin release are not clear, and the high frequency firing of orexin neurons may trigger the release of orexin from dense core vesicles.^[13,14]^ In addition, as with other spontaneously released neuropeptides, some basal levels of orexin tone may be present all time; in support of this, orexin receptor antagonists promote sleep in humans even at night, when orexin neurons might be expected to be inactive.^[16]^

#### 1.2.2. Impact of orexin loss.

##### 1.2.2.1. Poor ability to stay awake.

Almost all patients with narcolepsy feel sleepy during the day and are prone to transition to Non-REM sleep.^[17]^ However, the drowsiness of most patients can be relieved after waking up in the morning or taking a nap, indicating that the symptoms are recoverable. But drowsiness returned after just an hour or two, indicating that the system that maintains wakefulness is dysfunctional. When immobilized or encouraged to sleep, narcoleptics can fall asleep very quickly.^[18]^ Patients were instructed to attempt sleep every 2 hours on a multiple small sleep latency test (Multiple Sleep Latency Test). On average, narcoleptics fall asleep in less than 8 minutes out of 5 20-minute sleep opportunities, and they tend to take only 1 to 2 minutes, while people without narcoleptics usually take 10 to 20 minutes.^[19]^ Moreover according to related experiments, due to the frequent and rapid transition to non-REM, the awake time in orexin-null mice and mice lacking orexin neurons was only half that of the control group.^[20]^

#### 1.2.3. REM sleep is poorly regulated.

Orexin neurons inhibit REM sleep, and patients with narcolepsy exhibit dysregulation of REM sleep as manifested by poor circadian rhythm of REM sleep, rapid transition to REM sleep and disruption of REM physiology. Normally, REM sleep occurs only during typical sleep hours, but because of the loss of the orexin signal, REM sleep can occur at any time of day.^[21]^ In fact, this pattern is central to the diagnosis of narcolepsy. In Multiple Sleep Latency Test, narcoleptics typically enter REM sleep during 2 or more of their 5 daytime naps, whereas healthy people rarely enter REM sleep during the day.^[22]^ In fact, REM sleep is usually preceded by at least 60 minutes of non-REM sleep at night, but in NT1, REM sleep often occurs within a few minutes of sleep onset.^[23]^

Mechanistically, the tendency of narcolepsy to enter REM sleep during the day may be due to uncontrolled circadian rhythms or inhibition of REM sleep.^[10]^ REM sleep exhibits a strong circadian rhythm. It is normally inhibited during the active phase by circadian signals transmitted from the suprachiasmatic nucleus to the dorsomedial hypothalamic nucleus (Dorsomedial Hypothalamic Nucleus, DMH).^[24]^ According to related studies, DMH neurons send signals to orexin neurons, and the absence of orexin neurons in mice reduces the amplitude of circadian sleep rhythm by half.^[25]^ These results suggest that orexin neurons, together with additional projections from the DMH, contribute to the suppression of REM sleep^[19]^ during the active phase.

#### 1.2.4. Evidence of autoimmune mechanisms.

Narcolepsy is caused by the selective destruction of orexin-producing neurons, so what kills orexin-producing neurons remains a major mystery. However, according to the latest research, there is a lot of evidence that NT1 is an autoimmune disease mediated by T cells.^[26]^ T cells can be divided into 2 categories, CD4 + helper T cells secrete cytokines to regulate or assist the active immune response. While CD8 + killer T cells use cytotoxic particles to lyse their target cells. Both reactive types of T cells are triggered through T cell receptors (TCRs) that recognize small, processed peptides presented to them by major histocompatibility complex (Major Histocompatibility Complex, MHC) molecules on antigen presenting cells; peptide fragments that bind to MHC molecules are called MHC-peptide complexes. CD4 + T cells recognize antigens^[26]^ that bind to MHC class II molecules on the surface of antigen-presenting cells. While CD8 + T cells respond to antigens presented by MHC class I molecules, which are expressed on all nuclear cells but rarely in neurons.^[27]^

#### 1.2.5. MHC class II alleles and susceptibility to narcolepsy.

human leukocyte antigen (HLA) alleles strongly influence the development of narcolepsy. More than 90% of NT1 patients carry HLA class II alleles DQB1*06:02,^[28,29]^ and crystal structure modeling shows that a fragment of the pre-orexin protein closely matches the binding groove of DQB1*06:02.^[30]^ The DQB1*06:02 allele confers a 200-fold increased risk of NT1,^[31]^ a known association of HLA with any disease-NT1 rarely occurs in people who lack this allele. Homologues of this allele have twice the risk of NT1 compared with heterogenors.^[32]^ The HLA allele DQAI*01:02 is in strong linkage disequilibrium with DQBI*06:02 and carries a similar risk.^[33]^ DPBI*05:01 also increases the risk of NT1, although the results have a smaller impact.^[34]^ In contrast, some other class II alleles had the greatest effect on narcolepsy risk, but 2 studies showed a small and independent effect of class I alleles.^[35]^

#### 1.2.6. Orexin neurons may be killed by t cells.

In addition to the above genetic evidence involving the MHC class II DQBI*06:02 allele, a specific polymorphism at the locus encoding the TCR alpha chain^[36]^ was included. Other evidence suggests that a T cell-mediated immune mechanism destroys orexin neurons in NT1 and that CD4 + cells are critical. It is important to note that CD4 + T cells may not directly destroy orexin neurons,^[37]^ but they can release cytokines that stimulate the attack on orexin neurons by CD8 + cells, macrophages, and natural killer cells. Using a transgenic mouse model in which orexin neurons express hemagglutinin and T cells possess a hemagglutinin-specific TCR, a research team showed that CD8 + cells are capable of destroying orexin neurons.^[38]^ However, the primary attack of CD8 + cells on orexin neurons cannot occur in NT1 because MHC class I molecules involved in CD8 + cell activation are rarely expressed in humans except during early development and after exposure to interferon-gamma.^[39]^

### 1.3. TCM treatment based on neurobiological mechanism

As mentioned above, according to the current Western medicine research, the occurrence of narcolepsy has a relatively clear link with the loss of orexin neurons, and according to the progress of TCM research, the basic pathogenesis of this disease is deficiency in origin and excess in superficiality, Yang deficiency and Yin excess. At this level, the research of Chinese and Western medicine can refer to each other and combine with each other. Therefore, the treatment of narcolepsy based on neurobiological mechanisms combined with TCM syndrome differentiation is feasible and worthy of study.

### 1.4. Progress of TCM research on narcolepsy

Although the basic pathogenesis is clear, the etiology and pathogenesis of narcolepsy are relatively complex, and there are relatively few studies on TCM. So far, the cognition of the etiology, pathogenesis, and syndrome types of narcolepsy has not been able to form a relatively unified cognition.^[40]^ Professor ZHang Lei^[41]^ divided the disease into 5 syndromes: Yang deficiency and mental stagnation, liver wind with phlegm, intertwined phlegm-blood stasis pattern, and deficiency of the spleen and kidney pattern. Professor ZHENG Zhongqi^[42]^ divided narcolepsy into 3 stages: deficiency of the liver and kidney pattern in the early stage, damp heat in the spleen and stomach pattern in the middle stage, and disharmony between the Ying nutrients and Wei-defence pattern in the late stage. Professor Wang Xuefeng^[43]^ treats narcolepsy from lung and spleen. In addition, Professor Qiu Changlin^[8]^ treated narcolepsy from spleen deficiency, and divided the disease into spleen deficiency with phlegm dampness pattern, Kidney yang deficiency pattern, kidney essence deficiency pattern and blood stasis due to qi stagnation. It is gratifying that increasingly TCM research teams have begun to break through the limitations of personal experience and use modern science and technology to study this disease systematically and normatively.

Most of the TCM syndrome types divided by the above TCM experts are less important in this article. Due to the limited space of this article, if the above TCM syndrome types have little to do with the article, they will not be discussed. Consult the literature and the WHO international standard terminology on TCM.

Treatment of narcolepsy is based on syndrome differentiation of TCM (Tables 1 and 2).

**Table 1:** Frequency distribution of traditional Chinese medicine.

Medication	Numbers	Frequency
Licorice	11	68.75%
Poria cocos	9	56.25%
Atractylodes macroce	7	43.75%
Rhizoma Acori Tatarinowii	5	31.25%
Ginseng	5	31.25%
Turmeric	4	25.00%
Bupleurum	4	25.00%
Cassia twig	3	18.75%
Peony	3	18.75%
Ephedra	3	18.75%
Polygala tenuifolia	3	18.75%
Astragalus	3	18.75%
Rhizoma Chuanxiong	3	18.75%
Ginger	2	12.50%
Big jujube	2	12.50%
Monkshood	2	12.50%
Radix Rehmanniae	2	12.50%
Morinda officinalis	2	6.25%
Fructus Aurantii Immaturus	2	12.50%
Yam	2	12.50%
Cortex Albiziae	2	12.50%
Semen Ziziphi Spinosae	2	12.50%
Scutellaria	2	12.50%
Pinellia Tuber	2	12.50%
Lotus seed heart	2	12.50%
Amomum villosum	2	12.50%
Platycodon grandiflorum	2	12.50%
Peach kernel	2	12.50%
Safflower	2	12.50%
Angelica	2	12.50%
Asarum	1	6.25%
Radix Rehmanniae Preparata	1	6.25%
Dogwood	1	6.25%
Fructus Evodiae	1	6.25%
Dendrobium	1	6.25%
Herba Cistanches	1	6.25%
Chinese magnoliavine fruit	1	6.25%
Radix Ophiopogonis	1	6.25%
Herba Agrimoniae	1	6.25%
Chinese prickly ash	1	6.25%
Pawpaw	1	6.25%
Bezoar	1	6.25%
Radix Pseudostellariae	1	6.25%
Flos Magnoliae	1	6.25%
American Ginseng	1	6.25%
Jiao Sanxian	1	6.25%
Keel	1	6.25%
Oysters	1	6.25%
Antelope Horn	1	6.25%
Uncaria	1	6.26%
Scorpio	1	6.25%
Lapis Chloriti	1	6.25%
Tangerine Peel	1	6.25%
Bamboo shavings	1	6.25%
Polygonum multiflorum	1	6.25%
Alpinia oxyphylla	1	6.25%
Prunella vulgaris	1	6.25%
Gardenia	1	6.25%
Borneol	1	6.25%
Costustoot	1	6.25%
Lentils	1	6.25%
Semen Coicis	1	6.25%
Fructus Aurantii	1	6.25%
Radix Cyathulae	1	6.25%
Cortex Lycii	1	6.25%
Red peony root	1	6.25%
Dangshen	1	6.25%
Mint	1	6.25%

**Table 2:** Frequency distribution of traditional acupoints.

Acupoint	Numbers	Frequency
Baihui	5	100.00%
Neiguan	2	50.00%
Shenting	2	40.00%
Ophryon	2	40.00%
Sanyinjiao	2	40.00%
Shenmai	2	40.00%
Zhaohai	2	40.00%
Fengchi	2	40.00%
Tianzhu	2	40.00%
The Four-Shen Points	1	20.00%
Suliao	1	20.00%
Sishengcong	1	20.00%
Shenmen	1	20.00%
Benshen	1	20.00%
Fengfu	1	20.00%
Shuigou	1	20.00%
Shangxing	1	20.00%
Changqiang	1	20.00%
Zhibian	1	20.00%
Yangbai	1	20.00%
Jianjing	1	20.00%

The basic ways of TCM therapy of the disease are decoction, acupuncture, and massage, and the first 2 are the most important.

So far, there is no unified cognition of syndrome differentiation and typing of this disease, so this paper discusses the treatment of this disease based on syndrome differentiation and treatment according to the classification of syndrome elements and pathogenic factors on the basis of summing up the experience of predecessors. Lang Yi et al^[44,45]^ searched all the journals in China Journal Full-text Database (1979–2017) and Wanfang Database (1990–2017), standardized the narcolepsy syndromes that met the requirements, extracted the syndrome elements, and studied the narcolepsy syndromes and syndrome elements based on the literature. It can provide a reference and basis for further determining the standard of TCM syndrome differentiation and classification of narcolepsy.

### 1.5. Deficiency syndrome

Deficiency in origin and excess in superficiality is the basic pathogenesis of this disease, so deficiency syndrome is also an important syndrome type of this disease, and the deficiency of this disease is different types such as kidney yang deficiency pattern, kidney essence deficiency pattern, deficiency of the spleen and lung pattern, and deficiency of the spleen and kidney pattern. Kidney Yang deficiency pattern is characterized by cold intolerance. Cold limbs (especially below the lower back and knee joints), a bright, pale or dark complexion, profuse, clear urine, and frequent urination at night. The tongue is pale. The pulse is weak. This pattern often occurs when kidney yang fails to warm the body. The syndrome of kidney deficiency and marrow deficiency has been discussed above. Lung system is a functional system composed of the lung, large intestine, skin, body, hair, nose, and lung meridian. Deficiency of the spleen and lung pattern is characterized by low-pitched cough with thin, clear sputum, shortness of breath, panting, low food intake, abdominal bloating, and loose stools. The tongue is pale with a white, slippery coating. The pulse is weak. This pattern often results from qi deficiency of the lung and spleen. Deficiency of the spleen and kidney pattern is characterized by low food intake, abdominal bloating, loose stools, low back soreness/pain, and tinnitus. This pattern often occurs as a result of deficiency of the spleen and kidney.

It should be noted that these syndromes are not distinct from each other, but often influence and interact with each other. Professor Zhang Lei^[41]^ is good at treating kidney yang deficiency pattern with Cinnamon branch and cinnamon soup (cassia twig, Chinese herbaceous peony, glycyrrhiza, ginger, and jujube) and Ephedra asarum aconite soup (ephedra, herba asari, and monkshood). Decoction of Rehmanniae (prepared rehmannia root, morinda officinalis how, dogwood, dendrobe, cistanche, monkshood, fructus schizandrae, cassia twig, poria, dwarf lilyturf root, acorus tatarinowii and polygala amflra) and Sijunzi Decoction (ginseng, poria, atractylodes macrocephala koidz and liquorice) were used to treat deficiency of the spleen and kidney pattern. Professor Qiu Changlin^[41]^ advocated that Poria & Cassia Combo Decoction should be used to treat kidney yang deficiency pattern and Dihuang Yinzi should be used to treat kidney essence deficiency pattern. Feng Fan, et al^[46]^ created Xingshuian Prescription, which used Agrimonia Pilosa, Ephedra, Morinda officinalis, Zanthoxylum bungeanum, Chaenomeles sinensis, Curcuma aromatica and Calculus Bovis in order to tonify deficiency and strengthen, refresh and induce resuscitation. 46 patients with narcolepsy were treated with 1 dose per day. The short-term efficacy was evaluated after 6 months of treatment, and the long-term efficacy was evaluated after 6 months of withdrawal. The effective rate of symptoms after treatment was observed. The results showed that the total effective rate was 95.7%. The total effective rate was 80.4%. After treatment, the number of cases with various symptoms was reduced, and the effective rate was more than 60.0%. It is concluded that the treatment of narcolepsy with Xingshuian Prescription can significantly improve the clinical signs and symptoms and has a good effect. Professor Wang Shaojie^[47]^ created a prescription for early administration, which was composed of Radix Astragali, Radix Pseudostellariae, Herba Ephedrae, Fructus Evodiae, Rhizoma Chuanxiong, Flos Magnoliae, Fructus Aurantii Immaturus, Rhizoma Atractylodis Macrocephalae, Poria and Radix Glycyrrhizae in order to Regulate qi and strengthen the spleen.

### 1.6. Qi stagnation pattern

Liver is a functional system composed of the liver, gallbladder, tendons, nails, and liver meridian. Qi stagnation pattern is characterized by migratory distension. Fullness and pain in the subcostal region and abdomen that alleviate after sighing, belching, bowel sounds, and flatus. The pulse is wiry. Often results from qi stagnation of the zang–fu organs or in the localized area.

Emotional discomfort and internal injury of the 7 emotions cause the Liver qi stagnation pattern, and cause Liver qi stagnation transforming into a fire pattern. Liver fire is generated in the middle, causing disharmony between the Ying-nutrients and Wei-defence, and causing stagnation in the upper part of the brain, resulting in drowsiness and excessive sleep. “Sheng Ji Zong Lu”^[48]^ records: “The liver and gallbladder are both solid, and Ying-nutrients and Wei-defence are blocked, then the pure one is turbid and disturbed, so he often sleeps and lies. Professor Wang Xuefeng^[49]^ is good at treating narcolepsy of the liver and regards “Spleen deficiency with qi stagnation pattern, and disharmony between the liver and spleen pattern" as the main pathological mechanism of the disease. She likes to use Radix Bupleuri, Radix Astragali, poria cocos, Rhizoma Atractylodis Macrocephalae, Rhizoma Dioscoreae, Radix Panacis Quinquefolii, Jiao Sanxian, Radix Curcumae, Albizzia julibrissin, Rhizoma Acori Tatarinowii. Professor Zheng Zhongqi,^[50]^ based on the 6 meridians syndrome differentiation of Shaoyang syndrome, considered that the disease was due to the stagnation of Shaoyang and the dysfunction of the cardinal, and used Xiaochaihu Decoction (Radix Bupleuri, Rhizoma Pinelliae, Radix Ginseng, Radix Glycyrrhizae, Rhizoma Zingiberis Recens and Fructus Jujubae) to reconcile Shaoyang, and soothe the liver and promote bile flow.

### 1.7. Wind syndrome

“Plain Questions”^[51]^ records: “Wind, good deeds and several changes.” Pathogenic wind is a pathogenic factor characterized by opening the skin pores, high mobility, and upward/outward movement. Paroxysmal sleep occurs suddenly, falls asleep, quickly wakes up as usual, and soon returns to sleepiness. It occurs frequently during the day, and there is no fixed number. The characteristics of this disease are similar to those of pathogenic wind. If emotional stimulation causes hyperactivity of the liver yang pattern and disturbs the spirit, it will occur during lethargy. Hyperactivity of liver yang pattern is characterized by dizziness of hyperactivity of liver Yang pattern, blurred vision, tinnitus, lower back pain, limb numbness, feverish sensations in the palms, soles and chest, flushed cheeks, restlessness, irritability, and a dry and bitter mouth. The tongue is red with a scanty coating. The pulse is thready and rapid. This pattern often occurs when liver yin fails to control liver yang. Qinggan Xingshen Decoction was prepared by Professor Zhang Lei.^[41]^ Cornu Saigae Tataricae, Ramulus Uncariae Cum Uncis, Scorpio, Radix Scutellariae, Lapis Chloriti, Rhizoma Pinelliae Preparata, Poria, Pericarpium Citri Reticulatae, Caulis Bambusae In Taenia, Fructus Aurantii Immaturus Preparata, and Radix Glycyrrhizae are used in the prescription to Soothe the liver and extinguish wind, then clear heat and transform phlegm. Professor Wang Shaojie^[47]^ treated the disease according to the theory of epilepsy and applied Fit-settling Pill to the treatment of the disease, which was taken in the evening with Radix Polygoni Multiflori Preparata, Radix Polygalae, Fructus Alpiniae Oxyphyllae, Plumula Nelumbinis, Rhizoma Acori Tatarinowii, Cortex Albiziae, Semen Ziziphi Spinosae, Radix Curcumae, Spica Prunellae, Fructus Gardeniae, Poria.

### 1.8. Damp phlegm pattern

In the treatment of this disease, pathogenic wind and phlegm dampness are closely related. It is recorded in Yi Xue Cong Zhong Lu^[52]^: “When the wind is born, it will carry the potential of wood and overcome the earth, and the earth disease will gather fluid and form phlegm.” Wind phlegm enters the meridians pattern, and the failure of consciousness leads to sudden lying and rapid sleep. Professor Zhang Lei^[41]^ considered that liver wind with phlegm was one of the syndromes of this disease, and the prescription was Qinggan Xingshen Decoction. Zhu Danxi^[53]^ believed that “all diseases are caused by phlegm,” and Song Xianyuan^[54]^ advocated the use of Raw Jujube Powder, which was made of raw jujube, Acorus gramineus and borneol, to Open the orifices and unblock impediments. Clinical observation of 18 patients with narcolepsy showed that 12 cases were significantly effective, 4 cases were effective, and 2 cases were ineffective. The total effective rate was 88.9%. The curative effect is quite good. Professor Qiu Changlin^[8]^ treated Phlegm dampness accumulating in the spleen pattern with Fragrant sand, 6 gentleman soup, and Shenlingbaizhu Powder based on this theory, strengthening the spleen and transform the dampness, inducing resuscitation and restoring consciousness.

### 1.9. Blood stasis syndrome

It is recorded in Plain Question “The heart is the ruler’s official, and the spirit comes from it.”^[55]^ The heart governs the blood and vessels, and the heart governs the bright spirit. If Seven emotions cause internal damage, it will lead to liver qi stagnation pattern. Qi stagnation leads to blood stasis. Blood stasis due to qi stagnation leads to obstruction of the heart vessels. Heart vessel stasis pattern leads to loss of disquieted heart, spirit pattern, dizziness, headache, drowsiness, and insomnia. Heart system is a functional system composed of the heart, small intestine, blood vessels, face, tongue, and heart meridian. The view of TCM is: Blood is a red liquid that circulates within the blood vessels to moisten and nourish the body. It is an essential substance to maintain life activities. Blood stasis is a pathological state of slow, coagulated or stagnant circulation of blood.

Wang Qingren’s famous prescriptions, Tongqiao Huoxue Decoction and Xuefu Zhuyu Decoction,^[56]^ should be used to treat this disease. Modern physicians have also made innovations on this basis. Cui yuanwu^[57]^ modified Xuefu Zhuyu Decoction (Radix Rehmanniae, Semen Persicae, Flos Carthami, Fructus Aurantii, Radix Bupleuri, Rhizoma Chuanxiong, Radix Platycodonis, Radix Cyathulae, Radix Angelicae Sinensis, Radix Glycyrrhizae Preparata, Cortex Lycii, Radix Paeoniae Alba) to treat this disease. Qiu Changlin^[8]^ used Zhenwu Decoction and Xuefu Zhuyu Decoction to treat narcolepsy. He liked to use Radix Aconiti Lateralis Preparata, Rhizoma Atractylodis Macrocephalae, Rhizoma Zingiberis, Ramulus Cinnamomi, Rhizoma Chuanxiong, Fructus Aurantii, Radix Angelicae Sinensis, Flos Carthami, Radix Polygalae, and Rhizoma Acori Tatarinowii. Chen Jinghe^[58]^ used Taoren Siwu Decoction (Semen Persicae, Flos Carthami, Radix Rehmanniae, Radix Paeoniae Alba, Radix Angelicae Sinensis, and Rhizoma Chuanxiong) as the main prescription for treating systemic diseases, and proposed fifteen methods for promoting blood circulation and removing blood stasis based on syndrome differentiation and treatment.

Treatment based on pattern identification of Acupuncture and Moxibustion in TCM.

According to relevant studies, acupuncture and moxibustion treatment of narcolepsy^[59]^ can activate the excitability of the cerebral cortex, while reducing the adverse reactions of drug treatment, combined with TCM decoction treatment, quick effect, good curative effect, can play a “simple and inexpensive” therapeutic effect.

According to the related research, the acupoint selection rule of acupuncture and moxibustion for narcolepsy,^[60]^ the most frequently used acupoint is Baihui, and the acupoints for this disease are mostly distributed in the head, face, neck and neck. Li Shizhen’s “Compendium of Materia Medica · Magnolia · Invention”^[61]^ says that “the brain is the house of the primordial spirit,” and acupuncture at the acupoints on the top of the head embodies the principle of “where the acupoints are, where the indications are.” Baihui is the meeting point of Yang. Acupuncture at this point can connect yin and Yang veins, run through the meridians and acupoints of the whole body, regulate the body, and achieve the state of Yin and Yang. The occurrence frequency of Neiguan is second only to Baihui. Neiguan belongs to the Pericardium Meridian of Hand-Jueyin and is a collateral point, which passes through the Yin and Wei Meridians and has the effects of calming the heart and tranquilizing the mind, regulating qi and relieving pain. Dacheng of Acupuncture and Moxibustion^[62]^ says: “At the junction of Jueyin” leads the way to Yin and Yang, open and block. Neiguan can harmonize Yin and Yang, calm the heart and tranquilize the mind, and make Yang come out from Yin, so that patients can get rid of lethargy and drowsiness, wake up and open the mind, and treat narcolepsy.

In the analysis of the frequency of using meridians,^[60]^ the frequency of using meridians from high to low was Governor Vessel, Bladder Meridian, Gallbladder Meridian, Stomach Meridian, and Spleen Meridian. The Governor Vessel is known as the “Sea of Yang Meridians,” which commands the Yang of the whole body and stimulates the Yang Qi of the whole body. The Classic of Difficulties: Twenty-eight Difficulties^[63]^ says: “The Governor Vessel starts” It belongs to the brain. The governor vessel ascends into the brain. When the governor vessel is full of qi and blood, it ascends into the brain. When the sea of marrow is full, the mind is clear. Bladder meridian, gallbladder meridian, and stomach meridian are all Yang meridians, which cooperate with the governor vessel to regulate and control Yang qi in the body, to achieve the effects of stimulating Yang qi, stimulating the mind, awakening the mind and inducing resuscitation, and dispelling restlessness and tiredness.

Professor Zhuang Lixing^[64]^ used acupuncture combined with medicine to treat narcolepsy. The main points were Sishenzhen, Shenting, Yintang, and Suliao, and the auxiliary points were Neiguan and Sanyinjiao. Radix Codonopsis, Poria, Rhizoma Atractylodis Macrocephalae, Radix Glycyrrhizae Preparata, Rhizoma Acori Tatarinowii, Radix Curcumae, Herba Menthae (added later), Radix Bupleuri, and Radix Astragali are used as TCMs. Zhao Yin^[65]^ and Ye Chenlin^[66]^ also advocated the combination of acupuncture and medicine for the treatment of this disease.

In addition, Zheng Hong et al^[67]^ Collected 32 patients with this disease by using the needling method of harmonizing Ying and Wei, with Shenmai and Zhaohai as the main points and Baihui, Sishencong, Fengchi, Shenmen, Shenting and Benshen as the matching points, and the total effective rate of treating this disease reached 87.5%. Ma Lixin et al^[68]^ selected Shenmai, Zhaohai, Baihui and Sanyinjiao by using the acupuncture method of tonifying Shenmai and purging Zhaohai. Regulating channels and collaterals, harmonizing Yin and Yang, stimulating Yang qi, refreshing, and inducing resuscitation, and treating the disease; Ci Qinren et al^[69]^ used filiform needle to prick Fengchi, Fengfu and Tianzhu first, then the purgative method was used to prick Shuigou and Yintang horizontally, and finally needling Neiguan and Shangxing to penetrate Baihui, in order to lift Yang Qi, restore consciousness and open orifices, and cured 18 patients with narcolepsy.

### 1.10. Massage and other treatment methods

In addition to acupuncture and medicine, there are massage, ear acupuncture, and other methods to treat the disease.^[40]^ However, due to their own limitations, these treatments are difficult to be used as the main treatment, more as an adjuvant treatment, combined with acupuncture and medicine, to treat narcolepsy. Ear acupuncture is a therapeutic method to stimulate points on the ear with an acupuncture needle.

Song Li^[70]^ and others mainly used massage techniques to instruct patients to lie on the right side, start from Changqiang point, pinch up the skin and subcutaneous tissue, push along the Governor Vessel to Baihui point, then push from Zhibian point to Tianzhu point along the Bladder Meridian, and from Yangbai point to Jianjing point along the Gallbladder Meridian, then release the soft tissue along both scapulae, lateral waist and sacrum with heavy manipulation of levator muscle and shaking separation, combined with electroacupuncture. 32 patients were treated and the effective rate was 93.7%.

Zhu Huiming et al^[71]^ treated 32 cases of narcolepsy with electroacupuncture combined with auricular point sticking, and the total effective rate was 85%. Ma Songtao^[72]^ also treated the disease with auricular point sticking alone.

## 2. Discussion

In recent years, the research on narcolepsy in TCM and western medicine has made rapid progress and achieved gratifying results, such as the study of T cells. Although many achievements have been made, the pathogenesis of this disease in Western medicine has not yet been fully understood. At present, it is clear that the pathogenesis of NT1 is due to the severe loss of orexin neurons, and the CSF orexin level of this disease is very low or almost undetectable. However, the neurobiological mechanism of NT2 is not yet fully understood, and 90% of NT2 patients have normal or exin levels. NT2 affects up to half of narcoleptics, but unfortunately little is known about it.

TCM research on this disease started late, but under the guiding ideology of TCM holistic concept and syndrome differentiation and treatment, it has also made considerable progress by drawing on the valuable experience of ancient masters. However, there are still many problems in the study of this disease in TCM.

Through the collation of narcolepsy syndrome literature, it is found that there are still many problems in the study of TCM syndrome of narcolepsy. Because narcolepsy is a rare disease, the sample size is limited, most of the literature is small sample research, the syndrome type is single, which limits the quality of research, and still needs a larger sample size of high-quality syndrome research. Moreover, most of the literatures lack the description of syndrome differentiation standards, and most of them have no standards or self-made standards, and the disunity of syndrome differentiation standards also limits the reliability of research conclusions. Even though some studies have adopted clear and recognized criteria for syndrome differentiation, such as national standards, diagnostics of TCM, etc., because the above criteria are the criteria for syndrome differentiation of the whole disease domain, the 4 core symptoms of narcolepsy, namely, paroxysmal somnolence, sudden onset, sleep paralysis and hallucination, are not included in the criteria of syndrome differentiation, and only the peripheral symptoms are used as the basis for syndrome differentiation. It is easy to cause bias in syndrome differentiation. The establishment of standardized syndrome diagnostic criteria for narcolepsy is the premise and basis for in-depth study of TCM and multicenter collaborative research of this disease. This study initially reflects the distribution law of narcolepsy syndromes and syndrome elements, which can provide a reference and basis for further determining the TCM syndrome differentiation criteria of narcolepsy.

Because of the above reasons, the author of this article has to admit with regret that there are few high-quality randomized controlled trials and meta-analysis studies of narcolepsy in China and even in the world, which is one of the unsolved problems in the field of TCM so far, and this is also one of the purposes of writing this review, in order to inspire future researchers of TCM. In strict accordance with modern medical research design methods, a unified standard of TCM syndrome differentiation was formulated, to carry out high-quality randomized controlled trials and Meta-analysis studies.

Therefore, about this disease, Chinese medicine itself has advantages, can be combined with the frontier progress of Western medicine, forming TCM therapy the unique curative effect of narcolepsy, Western medicine for the pathogenesis of this disease is not yet clear, Chinese medicine can start from itself, form a diagnosis and treatment model with Chinese characteristics, combined use of TCM and Western medicine, treatment of narcolepsy.

It is believed that, with the continuous advancement of narcolepsy research, Chinese medicine will make increasingly progress, achieve increasingly gratifying success, and continue to contribute to the development of Chinese medicine.

In addition, because this article is a traditional literature review, on the one hand, based on the analysis of the literature that may include research results, the literature review article can cover a wide range of topics with different levels of completeness and comprehensiveness. But it must therefore be a very broad description. The literature review reviews the published literature, which means that the material included has some degree of permanence and has been through the peer review process. The literature review approach was designed to identify work and research that had been done previously and to build on previous work, but the literature review therefore lacked a clear intent to expand or analyze the data collected. Any conclusions that the authors themselves may draw are subject to bias, as they may inadvertently leave out important parts of the literature or fail to question the validity of the statements made. Objectively speaking, the TCM research on narcolepsy is still in a relatively scarce stage, and the content of clinical research and basic research is less, which makes the author face greater challenges in the process of comprehensive summary, and this article is bound to have some shortcomings, which is beyond doubt. However, the author hopes that this article will inspire more TCM researchers to enter this field and contribute to the research of this discipline. Therefore, the author here makes the most sincere apology to all experts and readers, and hopes that we will do better in future research.

## Author contributions

**Conceptualization:** Zhao Liu.

**Data curation:** Zhao Liu, Ruiqian Guan, Limin Pan.

**Formal analysis:** Zhao Liu.

**Funding acquisition:** Zhao Liu.

**Investigation:** Zhao Liu.

**Methodology:** Zhao Liu.

**Project administration:** Zhao Liu.

**Resources:** Zhao Liu.

**Software:** Zhao Liu.

**Supervision:** Zhao Liu.

**Validation:** Zhao Liu.

**Visualization:** Zhao Liu.

**Writing – original draft:** Zhao Liu.

**Writing – review & editing:** Zhao Liu.
